# EGFR Signaling in Colorectal Carcinoma

**DOI:** 10.4061/2011/932932

**Published:** 2011-02-14

**Authors:** Alyssa M. Krasinskas

**Affiliations:** Department of Pathology, University of Pittsburgh Medical Center, Presbyterian Hospital, A610, 200 Lothrop Street, Pittsburgh, PA 15213-2546, USA

## Abstract

The epidermal growth factor receptor (EGFR) and its downstream signaling pathways are involved in the development and progression of several human tumors, including colorectal cancer. Much attention has been given to the EGFR pathway as of lately because both EGFR and some downstream components serve as targets for anticancer therapy. In addition to playing a critical role in targeted therapy, alterations in this pathway can have prognostic implications. The EGFR pathway and its impact on colorectal carcinogenesis and prognosis are the emphasis of this paper. Since prognosis is tightly related to response to various therapies, the predictive value of the components of this pathway will be briefly discussed, but this is not the focus of this paper.

## 1. Introduction

The epidermal growth factor receptor (EGFR) and its downstream signaling pathways regulate key cellular events that drive the progression of many neoplasms. EGFR is expressed in a variety of human tumors, including gliomas and carcinomas of the lung, colon, head and neck, pancreas, breast, ovary, bladder, and kidney. Mutations, gene amplification, and protein overexpression of various elements of this pathway not only contribute to carcinogenesis but also impact prognosis and provide specific targets for therapeutic intervention. The importance of EGFR and its signaling pathway in colorectal carcinogenesis is the topic of this paper. Since prognosis is tightly related to response to various therapies, the predictive value of the components of this pathway will be discussed, but only briefly. There is another paper in this series, “Impact of *KRas* mutations on management of colorectal cancer” by Sullivan and Kozuch, which provides an in-depth review of the predictive value of *KRas* and other members of the EGFR signaling pathway.

## 2. EGFR and the EGFR Signaling Pathway

EGFR is a 170-kDa transmembrane tyrosine kinase receptor that belongs to the ErbB family of cell membrane receptors. In addition to EGFR (also known as HER1 and ErbB-1), other receptors in this family include HER2/c-neu (ErbB-2), Her 3 (ErbB-3), and Her 4 (ErbB-4). All of these receptors contain an extracellular ligand-binding region, a single membrane-spanning region, and a cytoplasmic tyrosine-kinase-containing domain. 

**Figure 1:**
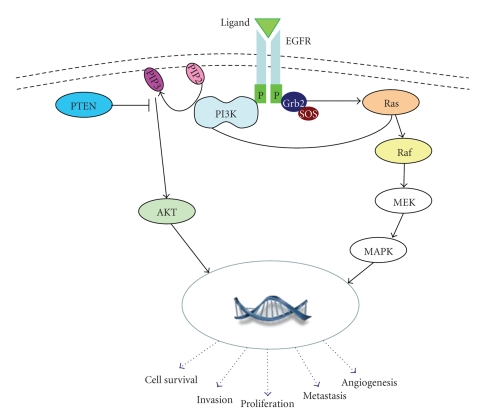
EGFR signaling pathway. Ligand binding induces dimerization and activates the EGFR. Subsequent autophosphorylation of tyrosine residues activates downstream signaling. In the Ras-Raf-MEK-MAPK, one axis of the EGFR signaling cascade, an adaptor protein complex composed of growth factor receptor-bound protein 2 adapter protein (Grb2), which harbors a tyrosine phosphate-docking site, and son of sevenless (SOS), a Ras GDP/GTP exchange factor, then activates the Ras GTPase. After activation, Ras (i.e., KRas) recruits and activates the serine protein Raf (i.e., B-Raf), and subsequent phosphorylation and activation of MEK and then MAPK occurs, resulting in activation of transcription factors in the cell nucleus. The Ras-Raf-MAPK signaling pathway is thought to control cell growth, differentiation, and survival (?apoptosis). The other axis of the EGFR signaling cascade that is important in colorectal carcinogenesis is the PI3K-AKT pathway. Once the EFGR tyrosine residues are phosphorylated, PI3K is translocated to the cell membrane and binds to tyrosine phosphate (through its adaptor subunit p85) which triggers the PI3K catalytic subunit p110 to produce phosphatidylinositol-3,4,5-triphosphate (PIP3). PI3K then promotes AKT activation. Activated AKT (p-AKT), present within the cytoplasm, then activates various targets that result in cell growth, proliferation, and survival (paralleling the Ras-Raf-MEK-MAPK signaling pathway). Importantly, these two axes are closely related and have some overlap. For example, the p110 subunit of PI3K can also be activated via interaction with Ras. Of note, phosphatase with tensin homology (PTEN) is a phosphatase that converts PIP3 back to phosphatidylinositol (4, 5) bisphosphate (PIP2), thereby negatively regulating the PI3K-AKT pathway.

In normal cells, the EGFR signaling cascade begins with ligand activation of EGFR (Figure 1). Up to eleven ligands can bind the ErbB family of receptors, including EGF and transforming growth factoralpha [1]. Ligand binding induces dimerization of the receptor with formation of homodimers and heterodimers, which leads to the activation of tyrosine kinase. The intracellular tyrosine kinase residues then become autophosphorylated, inducing activation of multiple signal transduction pathways. Two main intracellular pathways activated by EGFR are the mitogen-activated protein kinase (MAPK) pathway and the phosphatidylinositol 3-kinase- (PI3K-) protein kinase B (AKT) pathway. These pathways lead to the activation of various transcription factors that then impact cellular responses such as proliferation, migration, differentiation, and apoptosis [2]. 

Signaling through the EGFR pathway is a complex process that requires tight regulation [2]. The first level of complexity is encountered at the receptor level, where multiple ligands are shared and lateral signaling occurs between members of the ErbB family. Then there are positive and negative feedback loops built into the pathways and differential activation of transcription factors, depending upon the cell type. When this tightly regulated system goes awry, it can contribute to malignant transformation and tumor progression through increased cell proliferation, prolonged survival, angiogenesis, antiapoptosis, invasion, and metastasis [3, 4].

## 3. The EGFR Pathway and Colorectal Carcinogenesis (Table 1)

### 3.1. EGFR Protein Expression

EGFR expression (or overexpression), typically determined by immunohistochemistry, has been found to be associated with tumor progression and poor survival in various malignancies, such as carcinomas of the head and neck [5]. However, the significance of EGFR protein expression is controversial in other tumors, such as lung carcinomas [6]. Although EGFR has been reported to be overexpressed in anywhere from 25% to 82% of colorectal cancers [4], some recent studies report protein overexpression (defined as 2+ and/or 3+ staining or in >50% of cells) in 35 to 49% of cases [7–9]. However, the clinical significance of EGFR overexpression in colorectal cancer is uncertain. While one study of 249 colorectal cancers demonstrated an association of EGFR overexpression with tumor grade (poor differentiation) (*P* = .014) [8], another group found no association with grade in 134 tumors [9]. Similarly, some studies have found an association between EGFR overexpression (defined as 2+ or 3+ intensity) and reduced survival [7, 9], while others have not [4]. 

Due to the known expression of EGFR in colorectal cancer, a phase II trial of cetuximab, an anti-EGFR monoclonal antibody, in patients with refractory EGFR-positive (assessed by immunohistochemistry) colorectal cancer was undertaken [10]. The results of this trial, reported in 2004, were promising. It was soon discovered, however, that there was no correlation between EGFR expression in the tumor and response to therapy [11, 12]. In the study by Chung et al., four of 16 (25%) patients with EGFR-negative tumors who received cetuximab-plus-irinotecan therapy achieved a partial response with a greater than 50% reduction in the size of measurable lesions [11]. This response rate is nearly identical to the 23% response rate seen in a separate cetuximab-plus-irinotecan clinical trial in EGFR-positive patients [12]. As a result, cetuximab is now administered as indicated without the need for EGFR testing. 

The wide range of EGFR expression in colorectal cancer reported in the literature, as well as the uncertain significance of EGFR expression as a prognostic indicator, may be related to the methodology used to detect EGFR. Most studies use immunohistochemistry to detect EGFR expression in colorectal cancers. As demonstrated by the experience of HER2 expression in breast cancer, immunohistochemistry is highly dependent on the antibody clone that is used, staining protocols, selection of scoring methods, and selection of cutoff values. Until a standard method of EGFR staining and reporting is adopted, the significance of EGFR protein expression in colorectal cancer remains controversial.

### 3.2. EGFR Mutations, Gene Amplification, and Copy Number

Mutations affecting the extracellular domain of EGFR, often accompanied by gene amplification, are frequent in glioblastomas [13], while mutations in the tyrosine kinase domain of EGFR, also frequently associated with increased *EGFR* gene copy numbers, are clinically relevant in lung adenocarcinoma [6, 14–16]. Unlike lung cancer and other tumors, *EGFR* gene mutations are uncommon in colorectal cancers [17, 18]. 

The significance of *EGFR* gene amplification/increased *EGFR *copy number is more difficult to summarize. Some studies report that *EGFR* gene amplification (assessed by in situ hybridization methods) is uncommon in colorectal cancer [19, 20]. In contrast, in recent studies on chemorefractory colon cancers, it appears that modest increases in copy number (three- to fivefold) are present in up to 50% of cases [21]. It appears, however, that increased *EGFR* protein expression does not always translate into increased *EGFR* gene dosage [19, 21, 22]. For example, a study by Shia et al. found that only a small fraction (17 of 124 or 14%) of EGFR-positive (defined as 1+, 2+, or 3+) colorectal carcinomas detected by immunohistochemistry were associated with *EGFR* gene amplification (defined as >5 gene copies/nucleus) [19]. 

Similarly, the predictive significance of *EGFR* gene amplification is also confusing and uncertain. One study of 47 patients with metastatic colorectal cancer treated with a cetuximab-based regimen showed that EGFR gene copy gain, as assessed by fluorescence in situ hybridization, had no correlation with objective response rate, disease control rate, progression-free survival, or overall survival [23]. Conversely, another study of 173 patients with *KRas* wild-type metastatic colorectal cancer treated with a cetuximab-based regimen found that *EGFR* amplification/increased *EGFR* copy number, present in 17.7% of patients, was associated with response to anti-EGFR therapy [24]. These conflicting results may be related to the fact that there are no established guidelines for EGFR gene amplification. But since there are no guidelines, testing for EGFR gene amplification in colorectal cancer is not routinely performed. 

In addition to molecular alterations of the *EGFR* gene, activation of EGFR downstream effectors can lead to tumor formation/progression. Specific alterations can impact prognosis and predict response to anti-EGFR therapy.

### 3.3. KRas Mutations

The *KRas* proto-oncogene encodes a 21-kDa guanosine 5′-triphosphate- (GTP-) binding protein at the beginning of the MAPK signaling pathway. Somatic *KRas* mutations are found in many cancers, including 30%–40% of colorectal cancers, and are an early event in carcinogenesis [25–29]. *KRas* mutations, most commonly codon 12/13 missense mutations, lead to constitutive activation of the KRas protein by abrogating GTPase activity. These mutations result in unregulated downstream signaling that will not be blocked by antibodies that target the EGFR receptor.

The prognostic significance of *KRas* mutations is controversial. *KRas* mutation status is associated with shorter survival in some studies [28, 30–32], but not others [29, 32, 33]. The results of one study, which showed increased mortality with codon 13 G-A mutations but not with *KRas* mutations in general, suggest that prognosis may be related to specific mutations in the *KRas* gene [28]. Although not predictive of outcome with standard chemotherapy, *KRas* mutation status is a strong predictive marker of resistance to EGFR-targeted therapy in patients with metastatic colorectal cancer (i.e., *KRas *mutations predict a lack of response to anti-EGFR monoclonal antibodies cetuximab and panitumumab) [34–39]; this topic is discussed in detail in another paper in this series, “Impact of *KRas* mutations on management of colorectal cancer” by Sullivan and Kozuch.

### 3.4. BRAF Mutations

The *BRAF* gene encodes a serine-threonine protein kinase that is downstream of KRas in the MAPK signaling pathway. *BRAF* mutations occur in 5–22% of all colorectal cancers [40, 41]. When separated by microsatellite instability status, *BRAF* mutations are present in 40–52% of colorectal cancers that arise through the microsatellite instability pathway (MSI) pathway (microsatellite unstable tumors) [41–44], but only 5% of cancers are microsatellite stable [42]. The most frequently reported *BRAF* mutation is a valine-to-glutamic acid amino acid (V600E) substitution [45]. *BRAF* mutations are mutually exclusive with *KRas* mutations [41]. 

Unlike *KRas* mutations, *BRAF* mutations do have an impact on prognosis and survival. In some studies, the effect is dependent upon the microsatellite status of the colorectal cancer. Patients with a *BRAF* mutation in a microsatellite-stable colon cancer have significantly poorer survival than those without the mutation, but the *BRAF* status does not affect survival of patients with microsatellite-unstable tumors [29, 42]. In patients with metastatic *KRas* wild type tumors, *BRAF* mutations have been associated with shorter progression-free and shorter overall survival [24]. *BRAF* status also predicts response to anti-EGFR therapy. Of metastatic colorectal cancers that are found to be *KRas* wild type at codons 12/13, 5% to 15% can harbor *BRAF* mutations and show resistance to anti-EGFR therapy [46, 47]. The predictive role of *BRAF* mutations is further covered in another article in this series, “Impact of *KRas* mutations on management of colorectal cancer.”

### 3.5. The PI3K Pathway-*PIK3CA* Mutations and Expression of PTEN and p-AKT

The PI3K-AKT pathway can be deregulated by activating mutations in the *PIK3CA* gene (p110 subunit), by inactivation (often by epigenetic mechanisms) of the phosphatase and tensin homolog (*PTEN*) gene, or by activation of AKT [1, 48]. The *PIK3CA* gene encodes phosphatidylinositol 3-kinase (PI3K), a key signal transducer in the PI3K-AKT pathway. Mutations in *PIK3CA* occur in 14% to 18% of colon cancers, and most mutations involve hotspots on exons 9 and 20 [47, 49]. Interestingly, there is a strong association between *PIK3CA* exon 9 mutations and *KRas* mutations [47]. As a prognostic marker, *PIK3CA* mutations are associated with shorter cancer-specific survival, but this effect may be limited to patients with *KRas* wild-type tumors [49]. Briefly, as a predictive marker, only *PIK3CA* exon 20 mutations appear to be associated with worse outcome after cetuximab [47]. 

The* PTEN *gene encodes a protein tyrosine phosphatase enzyme (PTEN) that dephosphorylates phosphatidylinositol-3,4,5 triphosphate (PIP3) and thereby inhibits PI3K function [1]. Loss of PTEN results in constitutive activation of the PI3K-AKT pathway. *PTEN* mutations and loss of heterozygosity (LOH) of the *PTEN* locus have been reported in 13%–18% and 17%–19% of colon cancers, respectively [50, 51]. It appears that loss of PTEN has prognostic value. Loss of PTEN protein expression (assessed by IHC) is associated with shorter overall survival in patients with *KRas* wild-type tumors [24]. It appears that there is an association with *PTEN* mutations/LOH with MSI status, but the current published results are conflicting [50, 51]. PTEN protein inactivation may also be a negative predictor of response to anti-EGFR therapy [22, 52]. 

AKT is a major downstream effector of PI3K. A recent study by Baba et al. examined the role of activated (phosphorylated) AKT expression in a large cohort of colorectal cancers [48]. They demonstrated that p-AKT expression is associated with early stage disease and good prognosis. They also showed that p-AKT expression is associated with *PIK3CA* mutation, as expected from their relationship in the EGFR pathway, but that the prognostic effect of p-AKT expression was independent of *PIK3CA* mutation. It is possible that p-AKT expression could serve as positive prognostic marker in patients with colorectal cancer.

In summary, the EGFR signaling pathway is a complex and tightly regulated process that is involved in growth, proliferation, and survival of normal cells. When this system goes awry and unchecked, it can lead to growth, proliferation, survival, and metastasis of neoplastic cells. Alterations within the EGFR signaling cascade, such as gene mutations, gene amplification, and protein overexpression, have been shown to contribute to colorectal carcinogenesis. Some alterations also portend a poor prognosis in patients with colorectal cancer. Due to the complex interaction of EGFR and its downstream regulators, the study of individual components of this pathway often yields conflicting results, as noted in this paper. Hence, there are still many questions that need to be answered before we can fully understand the impact of the EGFR signaling pathway on colorectal carcinogenesis and the prognosis of patients with colorectal cancer.

## Figures and Tables

**Table 1:** Components of the EGFR signaling pathway important in colorectal cancer.

Component (gene/protein)	Protein function	Defect in CRC	Frequency	Impact	
				Prognostic	Predictive (to anti-EGFR therapy)
*EGFR*/EGFR	Transmembrane tyrosine kinase receptor	Protein expression	25–90%	Controversial	No correlation
		Mutation	Rare	Unknown	Unknown
		Increased copy number	0–50%*	Uncertain	Uncertain

*KRas*/KRas	GDP-/GTP-binding protein; facilitates ligand-dependent signaling	Activating mutation (codons 12, 13, 61, 146); leads to activation of MAPK pathway	30–40%	Controversial	No response (if *KRas* is mutated)

*BRAF*/B-Raf	Serine-threonine protein kinase downstream of KRas	Activating mutation (V600E)	5–12%	Poor prognosis in MSS tumors	No response (if *BRAF* is mutated)

*PIK3CA*/PI3K	A key signal transducer in the PI3K-AKT pathway	Activating mutation (exons 9 and 20)	14–18%	Poor prognosis in KRas wt tumors	No response (if exon 20 is mutated)

*PTEN*/PTEN	A protein tyrosine phosphatase enzyme; inactivates PI3K pathway	Loss of protein expression; mutation; LOH	13–19%	Poor prognosis in KRas wt tumors	No response (possibly)

CRC: colorectal cancer; LOH: loss of heterozygosity; wt: wild-type.

*Low % for high (>10 copies) amplification; higher % for low number of copies (3–5 copies).

## References

[1] Hynes NE, Lane HA (2005). ERBB receptors and cancer: the complexity of targeted inhibitors. *Nature Reviews Cancer*.

[2] Citri A, Yarden Y (2006). EGF-ERBB signalling: towards the systems level. *Nature Reviews Molecular Cell Biology*.

[3] Mitsudomi T, Yatabe Y (2010). Epidermal growth factor receptor in relation to tumor development: EGFR gene and cancer. *FEBS Journal*.

[4] Spano JP, Fagard R, Soria JC, Rixe O, Khayat D, Milano G (2005). Epidermal growth factor receptor signaling in colorectal cancer: preclinical data and therapeutic perspectives. *Annals of Oncology*.

[5] Chang SS, Califano J (2008). Current status of biomarkers in head and neck cancer. *Journal of Surgical Oncology*.

[6] Dacic S (2008). EGFR assays in lung cancer. *Advances in Anatomic Pathology*.

[7] Goldstein NS, Armin M (2001). Epidermal growth factor receptor immunohistochemical reactivity in patients with American Joint Committee on Cancer Stage IV colon adenocarcinoma: implications for a standardized scoring system. *Cancer*.

[8] McKay JA, Murray LJ, Curran S (2002). Evaluation of the epidermal growth factor receptor (EGFR) in colorectal tumours and lymph node metastases. *European Journal of Cancer*.

[9] Resnick MB, Routhier J, Konkin T, Sabo E, Pricolo VE (2004). Epidermal growth factor receptor, c-MET, *β*-catenin, and p53 expression as prognostic indicators in stage II colon cancer: a tissue microarray study. *Clinical Cancer Research*.

[10] Saltz LB, Meropol NJ, Loehrer PJ, Needle MN, Kopit J, Mayer RJ (2004). Phase II trial of cetuximab in patients with refractory colorectal cancer that expresses the epidermal growth factor receptor. *Journal of Clinical Oncology*.

[11] Chung KY, Shia J, Kemeny NE (2005). Cetuximab shows activity in colorectal cancer patients with tumors that do not express the epidermal growth factor receptor by immunohistochemistry. *Journal of Clinical Oncology*.

[12] Cunningham D, Humblet Y, Siena S (2004). Cetuximab monotherapy and cetuximab plus irinotecan in irinotecan- refractory metastatic colorectal cancer. *New England Journal of Medicine*.

[13] Frederick L, Wang XY, Eley G, James CD (2000). Diversity and frequency of epidermal growth factor receptor mutations in human glioblastomas. *Cancer Research*.

[14] Lynch TJ, Bell DW, Sordella R (2004). Activating mutations in the epidermal growth factor receptor underlying responsiveness of non-small-cell lung cancer to gefitinib. *New England Journal of Medicine*.

[15] Paez JG, Jänne PA, Lee JC (2004). EGFR mutations in lung, cancer: correlation with clinical response to gefitinib therapy. *Science*.

[16] Pao W, Miller V, Zakowski M (2004). EGF receptor gene mutations are common in lung cancers from "never smokers" and are associated with sensitivity of tumors to gefitinib and erlotinib. *Proceedings of the National Academy of Sciences of the United States of America*.

[17] Lee JW, Soung YH, Kim SY (2005). Absence of EGFR mutation in the kinase domain in common human cancers besides non-small cell lung cancer. *International Journal of Cancer*.

[18] Barber TD, Vogelstein B, Kinzler KW, Velculescu VE (2004). Somatic mutations of EGFR in colorectal cancers and glioblastomas. *New England Journal of Medicine*.

[19] Shia J, Klimstra DS, Li AR (2005). Epidermal growth factor receptor expression and gene amplification in colorectal carcinoma: an immunohistochemical and chromogenic in situ hybridization study. *Modern Pathology*.

[20] Spindler KL, Lindebjerg J, Nielsen JN (2006). Epidermal growth factor receptor analyses in colorectal cancer: a comparison of methods. *International Journal of Oncology*.

[21] Cappuzzo F, Finocchiaro G, Rossi E (2008). EGFR FISH assay predicts for response to cetuximab in chemotherapy refractory colorectal cancer patients. *Annals of Oncology*.

[22] Bardelli A, Siena S (2010). Molecular mechanisms of resistance to cetuximab and panitumumab in colorectal cancer. *Journal of Clinical Oncology*.

[23] Italiano A, Follana P, Caroli FX (2008). Cetuximab shows activity in colorectal cancer patients with tumors for which FISH analysis does not detect an increase in EGFR gene copy number. *Annals of Surgical Oncology*.

[24] Laurent-Puig P, Cayre A, Manceau G (2009). Analysis of PTEN, BRAF, and EGFR status in determining benefit from cetuximab therapy in wild-type KRAS metastatic colon cancer. *Journal of Clinical Oncology*.

[25] Burmer GC, Loeb LA (1989). Mutations in the KRAS2 oncogene during progressive stages of human colon carcinoma. *Proceedings of the National Academy of Sciences of the United States of America*.

[26] Leslie A, Carey FA, Pratt NR, Steele RJC (2002). The colorectal adenoma-carcinoma sequence. *British Journal of Surgery*.

[27] Brink M, de Goeij AFPM, Weijenberg MP (2003). K-ras oncogene mutations in sporadic colorectal cancer in The Netherlands Cohort Study. *Carcinogenesis*.

[28] Samowitz WS, Curtin K, Schaffer D, Robertson M, Leppert M, Slattery ML (2000). Relationship of Ki-ras mutations in colon cancers to tumor location, stage, and survival: a population-based study. *Cancer Epidemiology Biomarkers and Prevention*.

[29] Roth AD, Tejpar S, Delorenzi M (2010). Prognostic role of KRAS and BRAF in stage II and III resected colon cancer: results of the translational study on the PETACC-3, EORTC 40993, SAKK 60-00 trial. *Journal of Clinical Oncology*.

[30] Andreyev HJN, Norman AR, Cunningham D (2001). Kirsten ras mutations in patients with colorectal cancer: the ’RASCAL II’ study. *British Journal of Cancer*.

[31] Belly RT, Rosenblatt JD, Steinmann M (2001). Detection of mutated K12-ras in histologically negative lymph nodes as an indicator of poor prognosis in stage II colorectal cancer. *Clinical Colorectal Cancer*.

[32] Tejpar S, Bertagnolli M, Bosman F (2010). Prognostic and predictive biomarkers in resected colon cancer: current status and future perspectives for integrating genomics into biomarker discovery. *Oncologist*.

[33] Ogino S, Meyerhardt JA, Irahara N (2009). KRAS mutation in stage III colon cancer and clinical outcome following intergroup trial CALGB 89803. *Clinical Cancer Research*.

[34] Amado RG, Wolf M, Peeters M (2008). Wild-type KRAS is required for panitumumab efficacy in patients with metastatic colorectal cancer. *Journal of Clinical Oncology*.

[35] de Roock W, Piessevaux H, de Schutter J (2008). KRAS wild-type state predicts survival and is associated to early radiological response in metastatic colorectal cancer treated with cetuximab. *Annals of Oncology*.

[36] Di Fiore F, Blanchard F, Charbonnier F (2007). Clinical relevance of KRAS mutation detection in metastatic colorectal cancer treated by Cetuximab plus chemotherapy. *British Journal of Cancer*.

[37] Karapetis CS, Khambata-Ford S, Jonker DJ (2008). K-ras mutations and benefit from cetuximab in advanced colorectal cancer. *New England Journal of Medicine*.

[38] Lièvre A, Bachet JB, Boige V (2008). KRAS mutations as an independent prognostic factor in patients with advanced colorectal cancer treated with cetuximab. *Journal of Clinical Oncology*.

[39] Lièvre A, Bachet JB, Le Corre D (2006). KRAS mutation status is predictive of response to cetuximab therapy in colorectal cancer. *Cancer Research*.

[40] Garnett MJ, Marais R (2004). Guilty as charged: B-RAF is a human oncogene. *Cancer Cell*.

[41] Rajagopalan H, Bardelli A, Lengauer C, Kinzler KW, Vogelstein B, Velculescu VE (2002). RAF/RAS oncogenes and mismatch-repair status. *Nature*.

[42] Samowitz WS, Sweeney C, Herrick J (2005). Poor survival associated with the BRAF V600E mutation in microsatellite-stable colon cancers. *Cancer Research*.

[43] Domingo E, Laiho P, Ollikainen M (2004). BRAF screening as a low-cost effective strategy for simplifying HNPCC genetic testing. *Journal of Medical Genetics*.

[44] Loughrey MB, Waring PM, Tan A (2007). Incorporation of somatic BRAF mutation testing into an algorithm for the investigation of hereditary non-polyposis colorectal cancer. *Familial Cancer*.

[45] Davies H, Bignell GR, Cox C (2002). Mutations of the BRAF gene in human cancer. *Nature*.

[46] Loupakis F, Ruzzo A, Cremolini C (2009). KRAS codon 61, 146 and BRAF mutations predict resistance to cetuximab plus irinotecan in KRAS codon 12 and 13 wild-type metastatic colorectal cancer. *British Journal of Cancer*.

[47] de Roock W, Claes B, Bernasconi D (2010). Effects of KRAS, BRAF, NRAS, and PIK3CA mutations on the efficacy of cetuximab plus chemotherapy in chemotherapy-refractory metastatic colorectal cancer: a retrospective consortium analysis. *Lancet Oncology*.

[48] Baba Y, Nosho K, Shima K Phosphorylated AKT expression is associated with PIK3CA mutation, low stage, and favorable outcome in 717 colorectal cancers.

[49] Ogino S, Nosho K, Kirkner GJ (2009). PIK3CA mutation is associated with poor prognosis among patients with curatively resected colon cancer. *Journal of Clinical Oncology*.

[50] Zhou XP, Loukola A, Salovaara R (2002). PTEN mutational spectra, expression levels, and subcellular localization in microsatellite stable and unstable colorectal cancers. *American Journal of Pathology*.

[51] Nassif NT, Lobo GP, Wu X (2004). PTEN mutations are common in sporadic microsatellite stable colorectal cancer. *Oncogene*.

[52] Wilson PM, LaBonte MJ, Lenz HJ (2010). Molecular markers in the treatment of metastatic colorectal cancer. *Cancer Journal*.

